# Immunomodulatory role of vitamin D and emerging immunotherapies in hepatocellular carcinoma

**DOI:** 10.3389/fnut.2025.1611829

**Published:** 2025-09-17

**Authors:** Maurizio Chiriva-Internati, Fabio Grizzi, Marta Noemi Monari, Gianluigi Taverna, Jose A. Figueroa, Wei Daoyan, Robert S. Bresalier

**Affiliations:** ^1^Division of Internal Medicine, Department of Gastroenterology, Hepatology and Nutrition, The University of Texas MD Anderson Cancer Center, Houston, TX, United States; ^2^Department of Immunology and Inflammation, IRCCS Humanitas Research Hospital, Milan, Italy; ^3^Department of Biomedical Sciences, Humanitas University, Milan, Italy; ^4^Laboratory Analysis, Humanitas Mater Domini, Varese, Italy; ^5^Department of Urology, Humanitas Mater Domini, Varese, Italy; ^6^Oncology Consultants, Hurst Medical Clinic, Hurst, TX, United States

**Keywords:** cancer, vitamin D, HCC, liver, tumor microenvironment, vaccines, artificial intelligence, technology

## Abstract

Hepatocellular carcinoma (HCC) is one of the most common cancers globally, with nearly 1 million new cases diagnosed annually. It is a complex disease, with hepatitis B virus (HBV) and hepatitis C virus (HCV) infections being the most common etiological factors worldwide. Despite advances in therapy, survival rates for advanced and/or metastatic HCC remain low, with mortality rates 2.3 times higher in men than women. The liver’s immune system typically maintains an anti-inflammatory environment, contributing to immune tolerance to exogenous, food-derived antigens. However, disruption of the balanced interplay between immune factors within the hepatic microenvironment—due to viral hepatitis, excessive alcohol intake, non-alcoholic fatty liver disease (NAFLD) or non-alcoholic steatohepatitis (NASH)—can lead to chronic inflammation, oxidative stress, a cumulative mutational burden, cirrhosis, and eventually, malignant transformation. Once HCC is established, however, a functional pro-inflammatory immune response becomes critical to controlling tumor progression, as evidenced by the recent success of immune checkpoint inhibitor (ICI) treatments in HCC patients. In addition to ICIs, other novel immunotherapeutic intervention strategies, such as cancer vaccines and adoptive T cell therapies, are currently being investigated. Furthermore, adequate nutrition plays a critical role in modulating immune function, with vitamin D being a key nutrient for immune/regulation. In this review, we will discuss the potential role of vitamin D in HCC immunity and recent immunotherapeutic advances in the management of this malignancy.

## Introduction

Hepatocellular carcinoma (HCC) is the most common form of liver cancer in the world, with more than 1 million cases expected to be diagnosed in 2025 (1). HCC typically arises in patients with liver cirrhosis resulting from various underlying causes, including chronic hepatitis B (HBV) and hepatitis C (HCV) infections, alcohol-induced liver injury, metabolic dysfunction-associated steatotic liver disease (MASLD), and non-alcoholic steatohepatitis (NASH), among others. Of these, HBV and HCV infections account for the majority of HCC cases diagnosed worldwide, with NASH becoming the fastest growing etiology for HCC in western countries (2, 3). Furthermore, the Surveillance Epidemiology End Results (SEER) database has demonstrated that HCC has become the fastest growing cause of cancer-related death in the USA, illustrating its importance as a global health problem (4).

The liver has a complex and dynamic cellular immune microenvironment that includes combinations of cytotoxic, helper and regulatory T lymphocytes, myeloid-derived suppressor cells (MDSCs), dendritic cells (DCs), and natural killer (NK) cells (5, 6). The hepatic immune microenvironment tends to enhance self-tolerance, while an effective endogenous anticancer response depends on increased tumor infiltration of activated T cells, which promotes expression of tumor-specific antigens (TSAs) (7, 8). The balanced interplay between these cellular elements determines whether a tolerogenic or proinflammatory milieu permeates the hepatic parenchyma (9–11). For this reason, HCC is considered an immunogenic tumor and immunotherapy has become a promising treatment modality in this disease (12, 13).

One of the most valuable micronutrients, with regards to immune function, is vitamin D (14). Many populations around the world suffer from a deficiency in this essential vitamin, including Hispanic Mexican Americans, non-Hispanic African Americans, and individuals from various Middle Eastern countries (15). Additionally, low levels of this vitamin are more commonly observed in individuals with obesity and in older adults. Vitamin D is crucial for immune regulation, and its deficiency has been associated with a higher risk of developing HCC (16). Therefore, ensuring sufficient vitamin D levels may contribute to better clinical outcomes in patients with HCC.

**Figure 1:**
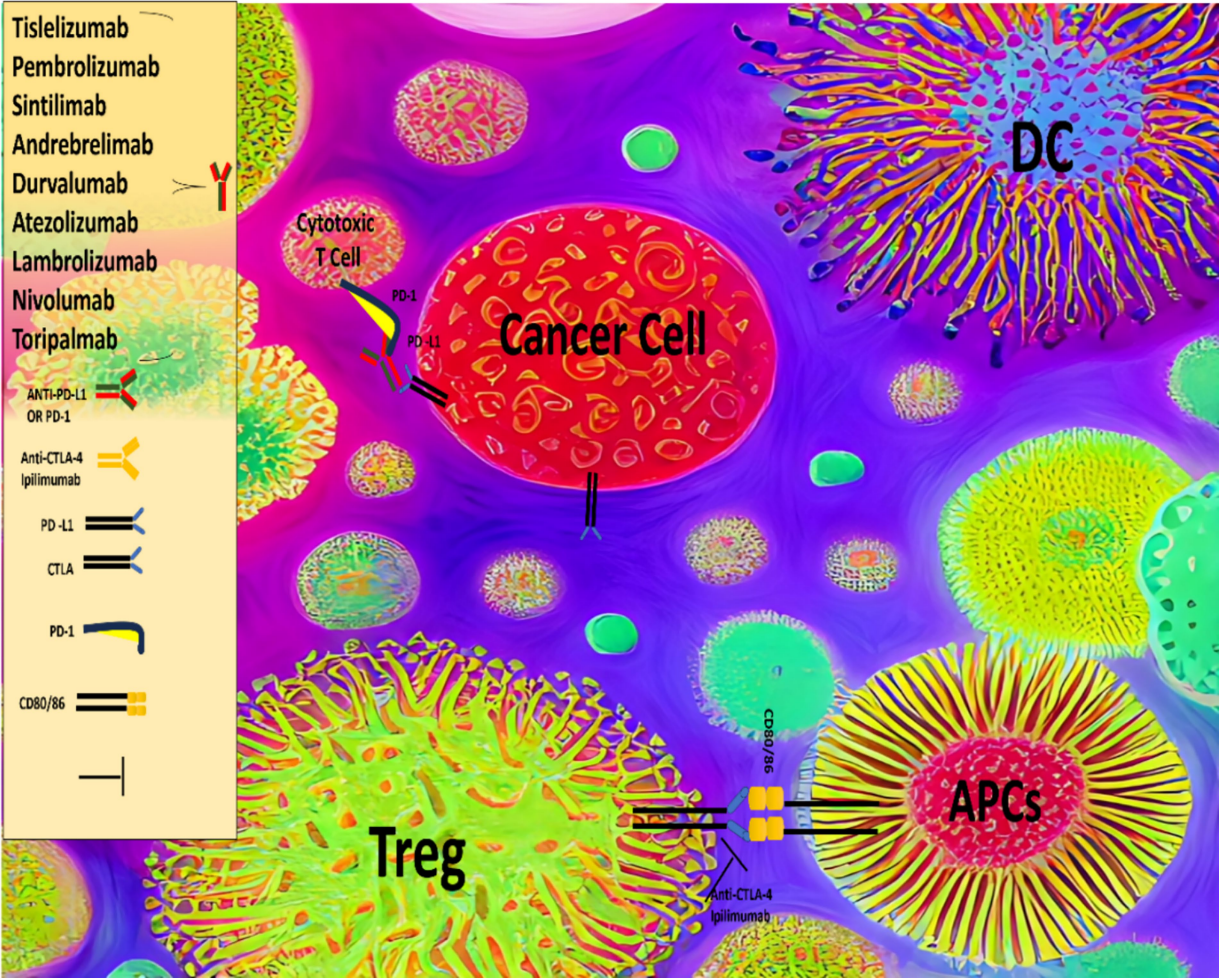
Comprehensive overview of the immune system, checkpoint inhibitors and HCC.

Given that HCC is widely regarded as a chemo-resistant malignancy, targeted therapeutic approaches have been developed, including tyrosine kinase inhibitors (TKIs) such as sorafenib, lenvatinib, regorafenib, and cabozantinib, as well as antiangiogenic agents like bevacizumab and ramucirumab. These therapies, whether used individually or in combination, have demonstrated significant clinical benefits, leading to their approval for the treatment of advanced HCC (17). More recently, several immunotherapeutic approaches have been investigated for the treatment of HCC, including the use of immune checkpoint inhibitors (ICIs) (Figure 1), vaccines, and adoptive T cell therapies, among others. ICIs, such as atezolizumab, nivolumab, pembrolizumab, durvalumab, ipilimumab and tremelimumab are antibodies that target regulatory immune checkpoints, including the cytotoxic T lymphocyte-associated antigen 4 (CTLA-4) and programmed cell death protein 1 (PD-1) pathways, and have shown substantial clinical efficacy in the treatment of advanced HCC (18, 19).

Cancer vaccines stimulate the immune system to recognize and attack cancer cells. They fall into two categories: prophylactic and therapeutic. Prophylactic vaccines prevent cancer by targeting cancer-causing viruses or bacteria, for example, the HPV vaccine prevents cervical and other HPV-related cancers (20). Therapeutic vaccines train the immune system to identify and destroy existing cancer cells (21–27). Despite their potential, cancer vaccines have yet to show consistent success in clinical trials. Adoptive cell therapies (ACT), using genetically engineered T cells, are also being investigated for HCC. Advances in molecular engineering have enabled the development of T cells modified to express chimeric antigen receptors (CARs) or transduced T cell receptors (T-TCRs) that specifically target tumor-associated antigens (TAAs) and TSAs (28–33). Key HCC TAAs include alpha-fetoprotein (AFP), viral antigens, and cancer-testis antigens like NY-ESO-1. Over 30 early-phase clinical trials of CAR-T and T-TCR therapies targeting HCC TAAs/TSAs are currently ongoing (34). Although still in early development, ACT strategies show significant promise for HCC treatment (35, 36). Additionally, the integration of data mining, machine learning, and artificial intelligence (AI) has enhanced the discovery of personalized immunotherapeutic targets, increasing the potential for novel HCC therapies (37). This review explores the evidence supporting vitamin D roles in cancer immunity and examines the impact of immunotherapeutic strategies in HCC management.

## Hepatocellular carcinoma and its immunological microenvironment

To manage the constant influx of intestinal antigens, the liver maintains a largely tolerogenic immune environment, unlike most organs (38). This complicates the interaction between malignant hepatocytes and hepatic immunity. HCC is often linked to chronic liver inflammation from factors like viral hepatitis, alcohol use, or NASH. Animal models show that pro-inflammatory cytokines, such as lymphotoxin-α, tumor necrosis factor-α (TNF-α), and interleukin-6 (IL-6), promote liver cancer and aggressive tumor traits (39, 40). Conversely, immune cell infiltration in tumors is associated with better outcomes, highlighting the role of immune activity in HCC progression and prognosis (41, 42). Chronic liver damage activates hepatic stellate cells (HSCs) (43), which respond by producing extracellular matrix proteins, including collagen and growth factors that drive endothelial cell migration, angiogenesis, and fibrosis, ultimately leading to cirrhosis and distorted liver architecture (44). HSCs also release transforming growth factor-β (TGF-β), which promotes fibrosis and suppresses cell proliferation (45). In addition, activated HSCs enhance immune tolerance by limiting lymphocyte infiltration, upregulating PD-L1, and promoting recruitment and differentiation of Tregs (46, 47). They also inhibit CD8^+^ T cell activation by disrupting IL-2 signaling and support the development of MDSCs (48, 49). In this altered environment, pre-malignant senescent hepatocytes secrete chemokines that weaken immune surveillance and promote immunosuppression (50). This tumor-permissive state in cirrhotic liver tissue is known as the “cancer field effect” and is linked to immune and inflammatory gene signatures that drive HCC development (51, 52). An immune-related cancer field signature has been found in up to 50% of adjacent cirrhotic tissue in HCC patients, classified into immunosuppressive or pro-inflammatory subtypes. The immunosuppressive type features strong TGFβ signaling, T cell exhaustion, high immune checkpoint expression, and increased HCC risk (53). Key innate immune cells play crucial roles in HCC development. Tumor-associated macrophages (TAMs) exist as tumor-suppressive M1 and oncogenic M2 types, both abundant in HCC. In co-culture, M1 TAMs promote cancer cell apoptosis and inhibit invasion, while M2 TAMs drive tumor growth and metastasis via pathways like TLR-4/STAT-3, TLR-4/NF-κB, and Wnt/β-catenin (40, 54). Tumor-associated neutrophils (TANs) also have immunosuppressive (N1) and oncogenic (N2) forms, with peritumoral TANs linked to HCC progression, increased PD-L1, and reduced T cells in non-alcoholic steatohepatitis (55). A lower neutrophil-to-T cell ratio in tumor tissue is linked to better patient survival, highlighting the prognostic value of TANs in HCC (56). MDSCs include myeloid progenitors and granulocytes that suppress NK cells and CD4^+^/CD8^+^ T cells, contributing to immune suppression in the HCC microenvironment (57). MDSCs inhibit cytotoxic T lymphocytes (CTLs) by producing inducible nitric oxide synthase (iNOS) and reactive oxygen species (ROS), disrupt dendritic cell development, impair NK cell function, reduce interferon-γ (IFN-γ) production, and promote T cell apoptosis via the Tim-3/Gal-9 pathway. They also stimulate Treg expansion by secreting immunosuppressive cytokines IL-10 and TGF-β (58, 59). Tregs further suppress immunity in HCC by inhibiting TNF-α and IFN-γ, reducing T cell proliferation and cytokine production (60). Notably, Treg accumulation around tumors correlates with disease progression and poorer survival (61, 62). In the healthy liver, NK cells are kept in a tolerogenic state due to inhibitory killer cell immunoglobulin-like receptors (KIRs) (63). During liver inflammation, circulating NK cells are recruited and activated by Kupffer cell-derived cytokines such as IL-2, IL-12, IL-15, and IL-18 (64). However, NK cell dysfunction has been observed in chronic liver diseases like NASH and viral hepatitis, contributing to an immunosuppressive tumor microenvironment (TME) (65–67). In HCC, reduced expression of NKG2D ligands, driven by TGF-β, has been linked to disease progression and early recurrence (68, 69). CTLs are key players in anti-cancer immunity in HCC and are linked to better prognosis (41). While CTLs are central to the adaptive immune response, some studies suggest their depletion reduces tumor burden, whereas others show they help surveil premalignant hepatocytes (70). Single-cell RNA sequencing reveals that CTLs in HCC are often dysfunctional (71), likely due to T cell exhaustion and the presence of immunosuppressive Tregs (62). Exhausted T cells show reduced proliferation, impaired cytokine production, decreased cytotoxicity, and increased expression of inhibitory receptors like CTLA-4, PD-1, LAG-3, and TIM-3 (72, 73). Additionally, CD4^+^ T helper 2 (TH2) cells, induced by IL-10 from tumor-infiltrating MDSCs, can inhibit CTL activity (74). Elevated TH2 cytokines (IL-4, IL-5, IL-10) are associated with HCC progression and metastasis (52). Animal and human studies suggest that B cells and tertiary lymphoid structures can both promote and inhibit HCC growth, depending on the model used (75–77). Notably, malignant hepatocytes secrete vascular endothelial growth factor (VEGF), which contributes to an immune-tolerant, tumor-promoting microenvironment in HCC (78, 79). Overall, interactions among TAMs, TANs, MDSCs, Tregs, CTLs, CD4^+^ T cell subsets, B cells, and malignant hepatocytes drive changes in the hepatic immune landscape, leading to a predominantly immunosuppressive environment that supports HCC development and progression (80). Thus, the pivotal role of the tumor immune microenvironment in HCC highlights the need to develop therapies that target specific molecular interactions between malignant hepatocytes and the immune system.

## Role of vitamin D on inflammation, immunity and management of HCC

**Figure 2:**
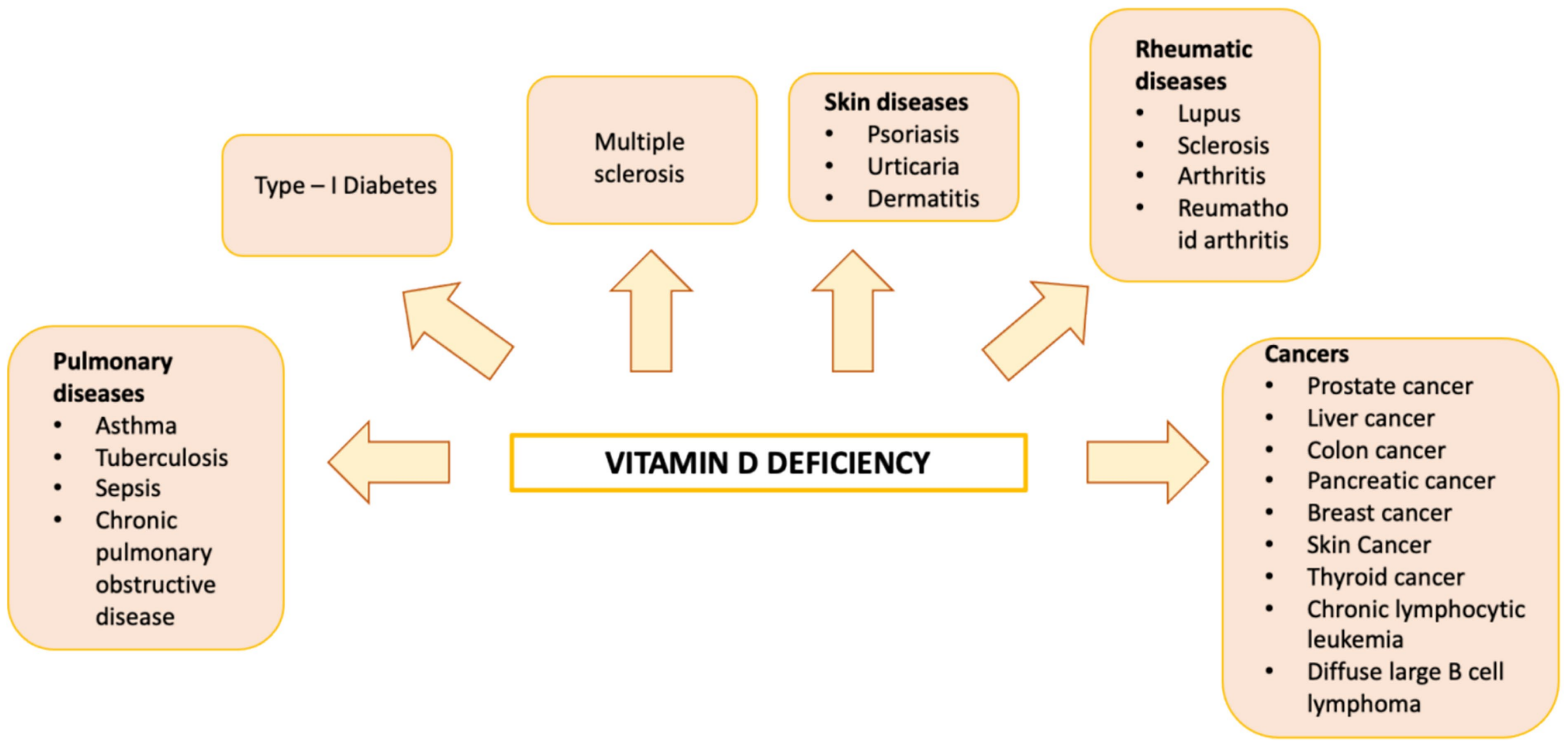
Diagram illustrating human diseases linked to vitamin D deficiency.

**Figure 3:**
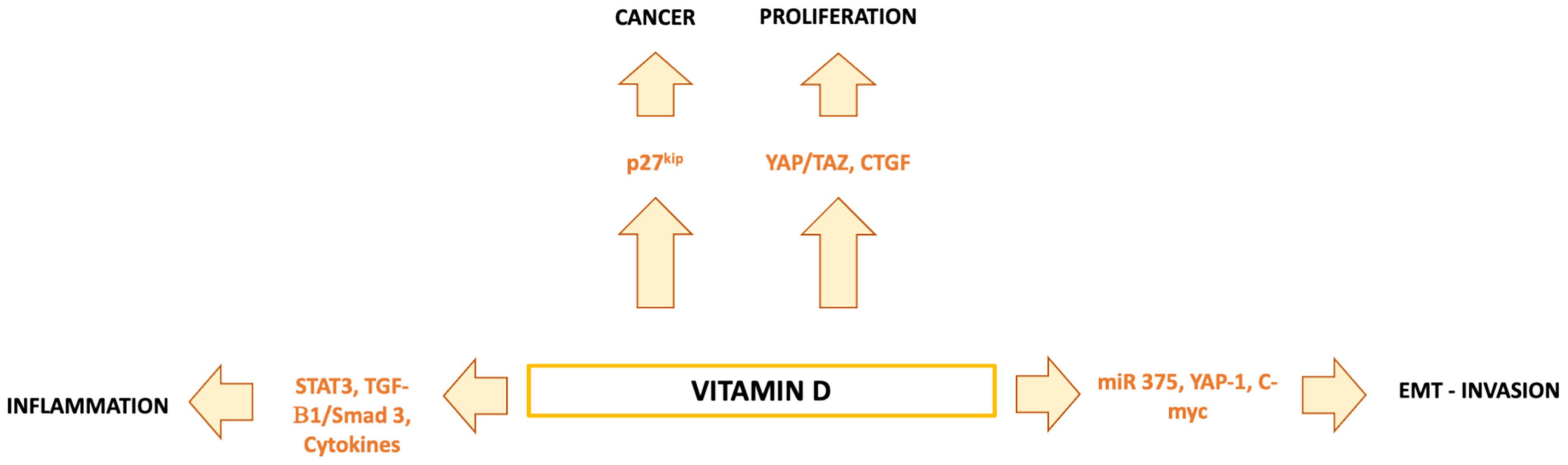
Vitamin D regulates hepatocytes, Kupffer cells, stellate cells, and liver cancer cells through VDR-dependent pathways. It reduces inflammation and stellate cell activation, while in HCC cells it exerts anti-proliferative, pro-apoptotic, anti-angiogenic, and pro-differentiative effects via multiple signaling cascades.

**Figure 4:**
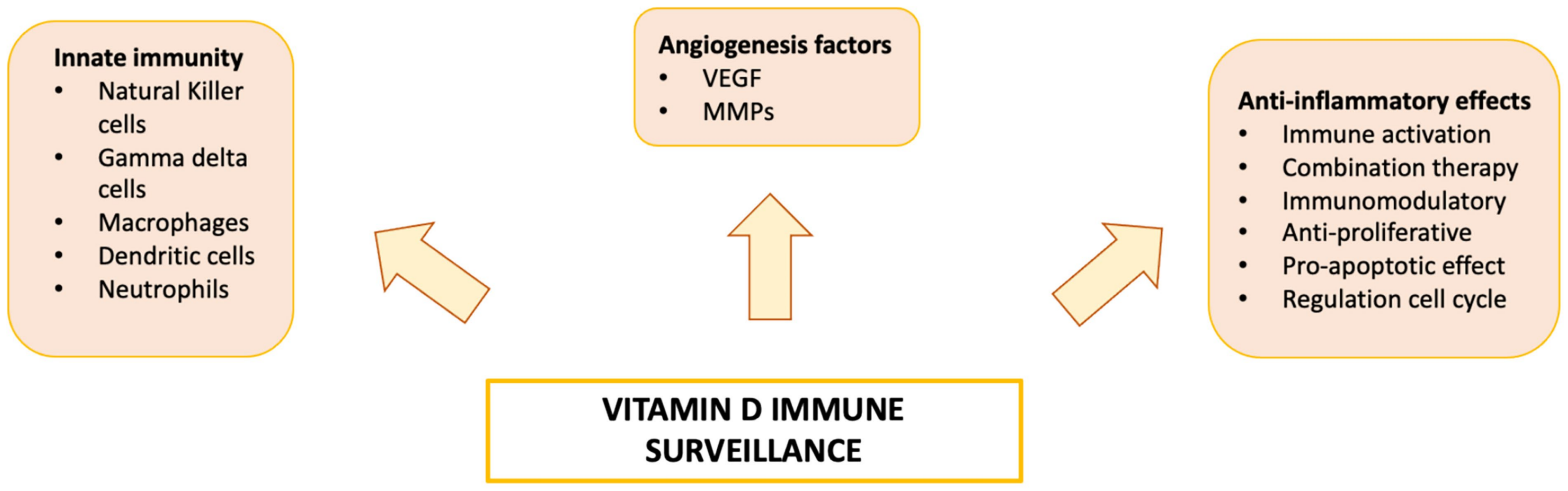
Vitamin D has been linked to impaired function of both the innate and adaptive immune systems, increasing susceptibility to the development and progression of various diseases.

Vitamin D is a fat-soluble nutrient essential for various physiological processes, including calcium and phosphorus absorption, immune regulation, and cell growth and differentiation. Although the underlying mechanisms are still being studied, evidence suggests that vitamin D deficiency contributes to the development of immune-related disorders and cancers, including HCC (81–90) (Figure 2). Vitamin D deficiency is influenced by factors such as geographic location, season, skin pigmentation, age, and lifestyle. It is more common in areas with limited sunlight, such as northern latitudes or regions with high pollution, and among populations with reduced sun exposure (91), older age, or darker skin pigmentation (81, 92). Vitamin D supports both innate and adaptive immunity by regulating inflammation and maintaining immune balance (84). Insufficient levels can disrupt this balance, potentially leading to chronic inflammation, autoimmune diseases, and cancer. The Institute of Medicine (IOM) recommends serum 25-hydroxy vitamin D (25(OH)D) levels of at least 20 ng/mL (50 nmol/L) for optimal bone health. Deficiency can be corrected through adequate sun exposure, diet, and supplementation if necessary (91). Vitamin D exists in two primary forms: vitamin D2 and vitamin D3. Vitamin D2 is derived from ergosterol and found in sources like yeast, mushrooms, and plants. Vitamin D3, on the other hand, is present in oily fish and produced in the skin from 7-dehydrocholesterol following sun exposure. Once in circulation, vitamin D3 is first converted in the liver by vitamin D-25-hydroxylase to 25-hydroxyvitamin D (25(OH)D). This is then further hydroxylated in the kidneys by 25-hydroxyvitamin D-1α-hydroxylase to produce the active form, 1,25-dihydroxyvitamin D3 (1,25(OH)₂D) (93, 94). The active form binds to the vitamin D receptor (VDR), forming a heterodimer with the retinoid X receptor (RXR). This complex binds to vitamin D response elements (VDREs) in DNA, regulating transcription of genes such as c-MYC and CDK1A. Beyond its genomic effects, 1,25(OH)₂D also exerts non-genomic actions that contribute to its wide range of biological functions. These non-genomic actions involve the activation of several intracellular signaling pathways, including phosphatidylinositol-3 kinase (PI3K), phospholipase C, phospholipase A2 (PLA2), and p21ras. These pathways lead to the production of second messengers such as cyclic AMP, calcium ions (Ca^2+^), and phosphatidylinositol 3,4,5-trisphosphate. These messengers, in turn, activate key protein kinases, such as mitogen-activated protein (MAP) kinases, protein kinase A (PKA), protein kinase C (PKC), Src, and Ca^2+^/calmodulin-dependent kinase II, and influence ion channel function, particularly calcium and chloride channels (95–98). Together, these mechanisms account for the rapid cellular responses observed with vitamin D activity. Vitamin D has drawn attention in liver cancer research due to its direct and indirect antineoplastic effects. It influences hepatocarcinogenesis by inhibiting hepatic stellate cells and Kupffer cells, and exerts direct anti-proliferative, anti-angiogenic, pro-apoptotic, and pro-differentiative effects on liver cancer cells. In the liver’s physiological context, hepatocytes normally express low levels of VDR; expression increases in NAFLD but decreases in NASH and chronic hepatitis C. VDR activation in hepatocytes has been linked to lipid accumulation and may contribute to steatosis. Kupffer cells, which abundantly express VDR, show reduced LPS-induced inflammation and downregulated IL-6, TNF, and IL-1β upon activation; VDR also mitigates ER stress–induced macrophage inflammation. Hepatic stellate (Ito) cells express significant VDR levels, and vitamin D or its analogs inhibit these cells via suppression of TGF-β/Smad signaling. In liver cancer cells, VDR is present in human HCC lines and patient samples, potentially regulated by KLF4. Vitamin D supplementation or analogs inhibit proliferation and induce apoptosis through multiple mechanisms: disruption of HGF/c-Met/ERK signaling; increased E-cadherin with reduced Akt; p27-mediated cell cycle arrest; HDAC2 reduction with p21 induction, modulating p53, Bax, DR5, caspase-8, and Bcl-2; regulation of TLR7 and β-catenin; and activation of TXNIP with Notch pathway inhibition, suppressing pro-inflammatory cytokines. These diverse pathways highlight vitamin D multifaceted role in modulating liver cell biology and the HCC microenvironment. Vitamin D modulates fibrosis by interfering with Smad binding, a signaling mechanism proposed to contribute to hepatic carcinogenesis (Figure 3). Inflammatory liver conditions such as alcoholic liver disease, viral hepatitis, NASH, and MAFLD can impair hepatic vitamin D metabolism by disrupting the synthesis of 25(OH)D. Vitamin D deficiency is commonly observed in these liver diseases (99, 100) and has been linked to an elevated risk of developing HCC compared to individuals with sufficient vitamin D levels (93). A meta-analysis by Yi et al. (101), which included 11 studies (6 case-control and 5 cohort) involving 12,895 participants, reported that vitamin D deficiency was associated with a significantly higher risk of HCC, supporting a potential protective role for vitamin D in liver cancer development. Similar findings were reported by Zhang et al. (82) in another meta-analysis involving 6,357 participants. The role of vitamin D in immune surveillance and regulation of angiogenesis in cancers, including HCC, remains an active area of investigation (Figure 4). Chronic inflammation, which promotes fibrosis, angiogenesis, and tumor progression, is a key factor in HCC development. Vitamin D has demonstrated anti-inflammatory effects in both HCC pathogenesis and within the tumor microenvironment (17, 102). In activated lymphocytes, 1,25(OH)₂D suppresses nuclear factor-kappa B (NF-κB) activity, leading to reduced production of interleukin-8 (IL-8), a pro-inflammatory cytokine essential for angiogenesis (103, 104). By dampening inflammation, vitamin D indirectly limits the formation of new blood vessels in HCC. Furthermore, vitamin D has been shown to reduce the expression of several angiogenic factors, including hypoxia-inducible factor-1 (HIF-1), vascular endothelial growth factor (VEGF), and IL-8, at both the protein and transcriptional levels (105, 106). Thus, vitamin D contributes significantly to maintaining liver health through its anti-inflammatory, anti-proliferative, anti-angiogenic, and pro-apoptotic properties, key mechanisms that help prevent the onset and progression of HCC (107). VDR is expressed in most immune cells, including B- and T-lymphocytes, monocytes, macrophages, and DCs, and some of these are able to convert 25-hydroxy vitamin D to 1,25 (OH)_2_D. For example, in the presence of infection, activated macrophages and monocytes, induced by TLR signaling and inflammatory cytokines, express CYP27B1 which converts 25(OH)D into 1,25(OH)_2_D. 1,25(OH)_2_D then enhances macrophages and monocyte antimicrobial activity by stimulating their production of endogenous cathelicidin via VDR signaling (108–110). Vitamin D also can affect neutrophils, eosinophils, and NK cells. Neutrophils express VDRs and their exposure to vitamin D influences their function by promoting the growth of neutrophil extracellular traps (NETs) and reducing inflammatory cytokine production (111). Vitamin D can also downregulate the expression of interleukin (IL)-15, a cytokine involved in the recruitment of eosinophils and NK cells, potentially impacting this aspect of innate immunity. Experimental studies have also suggested that differentiation, degranulation, cytokine secretion and cytotoxicity of NK cells can be modulated by 1,25(OH)2D, but available data are inconsistent (112–114). Macrophage production of 1,25(OH)_2_D not only regulates cathelicidin synthesis but can also influence lymphocyte function (93). Local production of 1,25(OH)_2_D by monocytes and macrophages results in a shift from proinflammatory to tolerogenic state through diverse mechanisms, including suppression of T cell proliferation and modulation of their cytokine production by promoting differentiation from helper T cell-1 (TH_1_) and helper T cell-17 (TH_17_) to a helper T cell-2 (TH_2_) immune subset (115–117). Similar to helper T cells, CTLs also express CYP27B1, and local conversion of 25(OH)D into 1,25(OH)_2_D can stimulate activation of VDR in response to infection and mitogenic stimuli (118–121). Furthermore, a decreased CD4^+^/CD8^+^ T cell ratio, an indicator of increased immune activation, has also been associated with low levels of 25(OH)D (122). It has also been reported that 1,25(OH)_2_D can modulate the differentiation and functions of antigen-presenting cells (APCs) by decreasing expression of major histocompatibility complex (MHC) class II and co-stimulatory molecules on the cell surface, in this way interfering with antigen presentation and IL-12 production, resulting in an immature and tolerogenic phenotype (123–127). 1,25(OH)_2_D can also promote Treg differentiation directly and via its interaction APCs, resulting in immunosuppression (128–130). Additionally, 1,25(OH)_2_D has been shown to suppress the expression of TLRs on monocytes, inhibiting the production of inflammatory cytokines such as IL-2, IL-6, and IL-17 (110, 131). 1,25(OH)_2_D can also decrease prostaglandin (PG) production through multiple pathways, including suppression of cyclooxygenase-2 (COX-2), an enzyme that catalyzes PG synthesis, increasing expression of 15-hydroxyprostaglandin dehydrogenase, (a catalyzer of PG degradation) and decreasing transcriptional regulation of PG receptors. This results in reduction of PG levels and inhibition of cell proliferation in several neoplasms, including breast, prostate and, possibly, liver cancers (132–134). Table 1 summarizes some of vitamin D effects of different components of the immune system. Besides its anti-inflammatory, antiangiogenic and tolerogenic immune effects, Vitamin D has been shown to exert direct antitumoral activities through several distinct mechanisms. For example, 1,25(OH)_2_D has been reported to increase expression of p21, p15, p16 and p27, all critical cyclin kinase (CDK) inhibitors and regulators of cell cycle kinetics and cell proliferation (135–140). 1,25(OH)_2_D has also been found to potentiate the function of KLF4, which upregulates VDR transcription, and thus forms a Vitamin D3-KLF4-VDR positive feedback loop, widely involved in induction of HCC cell differentiation, mesenchymal-epithelial transition (MET), inhibition of cell migration, invasion and proliferation (141), whereas loss of KLF4 expression is associated with advanced clinicopathological characteristics of HCC, significantly reduced CD8^+^ T cells and macrophage tumor infiltration, and predicts a poor prognosis for HCC patients (142–145). 1,25(OH)_2_D induces cell differentiation in colorectal cancer by repressing WNT/β-catenin signaling via several mechanisms, including increased nuclear export and decreased availability of β-catenin, increased expression of the WNT/β-catenin signaling inhibitor, Dickkopf-1 (DKK-1), and suppression of WNT/β-catenin signaling downstream targets (i.e., c-Myc and cyclin D) (146–149). All these mechanisms result in increased cell differentiation and decreased migration and invasion. 1,25(OH)_2_D3 can induce apoptosis through the mitochondrial pathway by suppression of the anti-apoptotic proteins (i.e., BCL-2 and BCL-XL) and induction of pro-apoptotic proteins (i.e., BAX, BAK and BAD) (150–156). 1,25(OH)_2_D has also been shown to potentiate the proapoptotic effects of certain chemotherapeutic agents in different malignancies, through decreased expression of ERK and AKT and upregulation of PTEN-mediated signaling (157–161). In breast cancer cells, 1,25(OH)_2_D has been reported to induce massive autophagy through calcium/calmodulin-dependent protein kinase kinase 2 (CAMKK2) activation of AMP-activated protein kinase (AMPK) (162). Additionally, 1,25(OH)_2_D promotes autophagy in irradiated, p53-expressing, breast and lung cancer cells, but not in p53-null cells, establishing the importance of this tumor suppressor in vitamin D-related autophagy (163, 164).

**Table 1:** Vitamin D modulates both innate and adaptive immunity by interacting with a variety of immune cell types and influencing key immune processes.

Cell type	Function	Effect
Dendritic cells	Cell maturation	Enhanced
Macrophages	Cell differentiation	Enhanced
APCs	Cell presentation	Reduced
Th1	Cell differentiation	Reduced
Th2	Cell differentiation	Enhanced
T-regs	Cell differentiation	Reduced
Th17	Decrease proliferation	Reduced
B cells	Decrease plasma cell generation	Inhibition of immunoglobulin secretion

It enhances innate defenses by promoting the antimicrobial activity of macrophages and dendritic cells, while also shaping adaptive responses by modulating T cell differentiation and inhibiting pro-inflammatory cytokine production.

## Therapeutic use of vitamin D in cancer: animal and human studies

Despite promising epidemiological and preclinical data indicating that 1,25(OH)₂D signaling may offer a preventive or therapeutic strategy against various cancers, including HCC, several challenges have limited confirmation in clinical settings. The primary concern is the risk of hypercalcemia and its associated adverse effects (165). A potential solution involves developing VDR agonists that replicate the anticancer effects of 1,25(OH)₂D without significantly raising serum calcium levels. However, current evidence supporting this approach remains limited and preliminary. In murine models of prostate and lung cancers, 1,25(OH)₂D treatment significantly reduced metastasis, partly due to its antiangiogenic effects (166, 167). It has also been shown to induce apoptosis and cell cycle arrest in tumor-associated endothelial cells without affecting normal tissues *in vivo* (168), suggesting a possible direct role in modulating endothelial function. However, definitive mechanistic evidence remains limited. In mice lacking renal 25(OH)D-1α-hydroxylase, the enzyme critical for producing active vitamin D, tumor development and heightened inflammatory responses were observed (169). Furthermore, in DEN-induced HCC models, loss of the vitamin D-upregulated protein 1 (VDUP1) led to increased tumor growth, enhanced cell proliferation, elevated TNF-α levels, and NF-κB activation, reinforcing the role of vitamin D–mediated pathways in hepatocarcinogenesis (170). *In vivo* studies with squamous cell carcinoma and prostate cancer, xenografts have shown that pre-treatment with 1,25(OH)₂D or its analogs enhances the antitumor effects of paclitaxel (157, 171). Similarly, 1,25(OH)₂D and analogs like paricalcitol and calcipotriol have been found to reduce MUC1, CD44, and GLUT1 expression, increasing pancreatic cancer cell sensitivity to gemcitabine *in vitro*. In gemcitabine-resistant pancreatic cancer mouse models, combining paricalcitol with gemcitabine significantly suppressed tumor growth (83). These findings suggest that vitamin D or its analogs may enhance the efficacy of cytotoxic therapies in certain cancers. The immune-regulatory function of vitamin D is well established, but its impact on cancer immunosurveillance and the efficacy of immunotherapy is still not fully understood. A recent study showed that mice with enhanced vitamin D availability exhibit enhanced immune-mediated resistance to transplantable cancers and improved responses to checkpoint blockade immunotherapies. In humans, a high vitamin D-VDR gene signature is associated with a lower cancer risk, improved survival, including HCC, and better responses to immune checkpoint inhibitors. In mice, this resistance is linked to vitamin D’s effects on intestinal epithelial cells, which shift the microbiome to favor *Bacteroides fragilis*, a bacterium that promotes cancer immunity. These findings reveal a previously overlooked link between vitamin D, gut microbial communities, and immune responses to cancer, suggesting that vitamin D levels may influence both cancer immunity and the success of immunotherapy (172). The vast majority of clinical trials exploring the safety and activity of 1,25(OH)_2_D, and its analogs, have been plagued by unreliable dosing, poor pharmacokinetic data, and few tumor responses (173–177). The vitamin D analog seocalcitol has created interest based on its ability to induce differentiation and growth inhibition in cancer cell lines and *in vivo*. Based on these findings, a total of 56 patients with advanced HCC were treated with oral seocalcitol for up to 1 year, with the dose titrated based on serum calcium levels. In this study, 2 out of 33 patients were reported to have complete tumor responses (CR) and 12 had stable disease, with one CR lasting at least 36 months at the time of report. Seocalcitol was well tolerated, with the main adverse effect reported being hypercalcemia and related symptoms (178). This study provided preliminary “proof of concept” for additional studies using vitamin D analogs in HCC. A phase II study by Beer et al. (179) combined a 0.5 μg/kg weekly oral dose of 1,25(OH)_2_D on day 1 with a weekly docetaxel dose on day 2, in patients with castrate resistant prostate cancer. No toxicities were reported and 30 out of 37 patients had a >50% reduction in PSA level. Based on these encouraging results the ASCENT 1 study randomized 250 prostate cancer patients to treatment with docetaxel ± 1,25(OH)_2_D. Investigators reported PSA response rates of 63% in the 1,25(OH)_2_D treated group and 52% in the placebo group (*p* = 0.07). Median overall survival (OS) was 24.5 months in the 1,25(OH)_2_D group and 16.4% in the placebo arm with a death hazard ratio of 0.67 (33% reduction in mortality, *p* = 0.04) (180). The confirmatory phase III study (ASCENT II) treated 900 prostate cancer patients with the same docetaxel/1,25(OH)_2_D plus prednisone combination, compared with a standard, higher dose docetaxel and prednisone, once every 3 weeks. Unfortunately, interpretation of ASCENT II was difficult due to a flawed design and the study was halted after the death rate in the experimental arm exceeded that found in the standard treatment group (107). Vitamin D interferes with cancer development and progression by downregulating inflammation, modulating angiogenic factor expression within the tumor microenvironment, and promoting cancer cell death through apoptosis and autophagy. These direct and indirect actions highlight the complex interplay between vitamin D, immune regulation, angiogenesis, and tumor proliferation. Further research is needed to better understand these mechanisms and their potential in developing targeted therapies for HCC. At the very least, ensuring sufficient serum vitamin D levels may help enhance the effectiveness of existing treatments in patients with HCC.

## Emerging immunotherapies in HCC

Systemic treatment of HCC is particularly challenging due to its intrinsic chemoresistance and the frequent presence of underlying liver dysfunction, factors that contribute to increased toxicity, limited treatment efficacy, and poor outcomes (17). As noted earlier, the hepatic microenvironment fosters immune tolerance, which enables HCC cells to evade immune detection. This immunosuppressive landscape has driven the development of immunotherapeutic approaches for HCC, including immune checkpoint inhibitors (ICIs) and engineered adoptive cell therapies that target TSAs associated with HCC.

## Immune checkpoint inhibitor therapy in HCC

Immune molecular checkpoints deliver inhibitory signals to different cellular components of the immune system and interfere with their ability to recognize and mount an effective response against cancer cells. The use of specific antibodies that block activation of these regulatory molecules associated with immune exhaustion (i.e., CTLA-4, PD-1 and PD1-L1), and known ICIs has rapidly extended the therapeutic armamentarium against HCC and other malignancies. The fully human CTLA-4 inhibitor, tremelimumab, was first tested in advanced, HCV-associated HCC patients, showing a response rate (RR) of 17%, a median time to progression (TTP) of 6.5 months and a probability of 1 year survival of 43% (181). Another study using tremelimumab in HCC patients reported resulting in 26% RR, TTP of 7.4 months and overall survival (OS) of 12.3 months (182). The clinical efficacy of PD-1/PD-L1 ICIs resides in its ability to augment the effector function of tumor-specific CTLs, resulting in cancer cell eradication (183). A phase I/II study of the PD-1 inhibitor nivolumab in 262 patients with advanced, HBV and HCV-infected and non-viral infected HCC, demonstrated a RR of 15 to 20% across dose escalation and OS of 15 months on the expansion cohorts (184). A follow-up phase III study comparing nivolumab with sorafenib as first-line therapy in unresectable HCC failed to show OS superiority of nivolumab over sorafenib. Pembrolizumab, a humanized anti-PD-1 monoclonal antibody, was evaluated as second-line therapy in 104 patients with advanced HCC, following sorafenib therapy. Investigators reported a RR of 17%, progressive-free survival (PFS) of 4.9 months, OS of 12.9 months and a 1-year survival of 54% (185). A phase III, placebo-controlled study of pembrolizumab in 413 previously treated HCC patients confirmed a RR of 16.9%, with a median duration of response of 13.8 months (186). No significant toxicities were reported in patients treated with nivolumab or pembrolizumab in these studies. Based on these results, both nivolumab and pembrolizumab received regulatory approval as second-line therapy in HCC following treatment with sorafenib. A phase I/II study using durvalumab, an anti-PD-L1 antibody, has reported RR of 10%, a median OS of 13.2 months and a 56% 1-year survival rate, with a favorable safety profile. The rationale behind combined CTLA-4/PD-1 blockade is based on the non-redundant biologic role of both pathways within the anticancer immunity cycle, with CTLA-4 being a driver of immune-suppression in APCs and Tregs and PD-1/PD-L1 acting predominantly as down-regulators of CTL response. The safety and efficacy of three different dosing schedules of the CTLA-4 inhibitor ipilimumab and nivolumab was evaluated in advanced HCC following prior sorafenib treatment at three different doses: nivolumab (1 mg/kg) + ipilimumab (3 mg/kg) or nivolumab (3 mg/kg) + ipilimumab (1 mg/kg) every 3 weeks for four doses followed by nivolumab maintenance (240 mg flat dose every 2 weeks), and nivolumab (3 mg/kg) + ipilimumab (1 mg/kg) every 6 weeks, until disease progression or toxicity. In this study the reported incidence of adverse events was 37% and RR was 31%. Following these results this combination received approval as second line treatment in advanced HCC. Durvalumab and tremelimumab combination treatment was also tested in a phase I/II study of 40 patients with advanced HCC, 70% with prior systemic treatment and 50% were not virally infected. Incidence of treatment-related AEs was 20%, RR was 15% and disease-control at 16 weeks was 57%. More recently a multicenter phase 1 clinical trial evaluated the effectiveness of atezolizumab (an anti-PD-L1 antibody) combined with bevacizumab (an anti-VEGF antibody) in the treatment of HCC (187). The results revealed a RR of 36%, the median OS of 17.1 months, and median PFS of 5.6 months for the combination, compared to atezolizumab monotherapy. A follow-up, global, phase 3 study compared treatment with atezolizumab and bevacizumab to sorafenib as a first line treatment for HCC (188). The RR for the atezolizumab–bevacizumab combination group was 27.3%, median PFS was 6.8 months and survival rates at 6 and 12 months were 84.8 and 67.2%, respectively, all statistically superior to sorafenib treatment. Based on these encouraging results, a combination treatment with atezolizumab with bevacizumab was recently approved as the preferred first-line treatment for advanced HCC. Results of these studies have changed the landscape of immunotherapy’s role in HCC and established ICI treatment as standard of care in this disease.

Evidence shows that in various cancers, including HCC, Vitamin D modulates immune responses and inflammatory processes within the TME by influencing both innate and adaptive immunity. Through these mechanisms, it may decrease immune tolerance to ICIs and synergistically improve their therapeutic efficacy. As above discussed, it is important to note that a wide range of immune cells, including T cells and macrophages, express both the VDR and 1α-hydroxylase. This dual expression endows these cells with critical functions: they can directly respond to vitamin D signaling, influencing immune responses, and they are also capable of locally activating vitamin D within tissues. Vitamin D modulates immune cells, reducing immunosuppression within the tumor microenvironment and thereby enhancing the therapeutic efficacy of ICIs. Vitamin D can increase CD8^+^ T cell activity, strengthen T cell cytotoxicity, and further improve the effectiveness of ICIs.

## Challenges associated with ICI therapy

Although ICIs are effective against many cancers, most patients eventually develop resistance. These therapies are costly and often inaccessible to patients in low-income regions or without insurance. Additionally, a small subset of patients experienced severe immune-related toxicities affecting organs like the colon, brain, skin, eyes, and liver, requiring immunosuppressive treatment. With new ICIs emerging, further research is needed to better understand their molecular mechanisms, resistance pathways, and ways to reduce side effects, particularly in HCC.

## Vaccine therapy in HCC

Cancer vaccine therapy aims to induce tumor control and/or sustained remission in cancer patients by promoting the detection, identification and eradication of malignant cells by the immune system, representing a promising immunotherapeutic strategy against HCC. As previously mentioned, cancer vaccines fall into two main categories: preventive and therapeutic. Preventive vaccines have shown efficacy in patients with established risk factors, such as viral infections, and vaccination against HBV and HPV has been successful in preventing HCC and cervical cancer, respectively. This preventive vaccination strategy may stimulate immune responses against early-stage malignancies, halting cancer progression in high-risk individuals (189). For example, a DNA vaccine against the B cell epitope GRP18-27, was reported to prevent hepatocarcinogenesis in the H22 murine hepatocarcinoma model (190). In another study, a TM4SF5 epitope-CpG-DNA-liposomal vaccine complex has been reported to induce immune memory against the target antigen in a mouse HCC model, suggesting its potential application in liver cancer prevention (191). A provocative study by Cai et al. (192) has demonstrated the use of a personalized neoantigen peptide vaccine could prevent postoperative HCC recurrences in patients with tumor vascular invasion, opening the door for further exploration of such an approach. Different from the goals of preventive vaccination, therapeutic cancer vaccines aim to stimulate a robust immune response against established malignancies that results in tumor control or remission. Therapeutic cancer vaccines encompassed a variety of designs and mechanisms of action to induce a cancer-specific immune response. These approaches involve the use of cancer-derived peptide and/or neoantigens, viral vectors, DNA, mRNA and APCs-based technologies (193). Given the multiple effects of vitamin D on the immune system, it is worth exploring how vitamin D, the VDR, and polymorphisms in vitamin D pathway genes may influence the adaptive immune response to vaccines. Emerging evidence identifies the gut microbiota as a critical regulator of immunotherapy response, while vitamin D, an immunomodulatory hormone, is gaining attention for its potential role in modulating both gut microbiota and immunotherapy outcomes. Vitamin D shows synergistic potential in cancer immunotherapy, primarily via the VDR, which is widely expressed in T cells, dendritic cells, and macrophages. VDR activation enhances antitumor immunity by promoting Treg differentiation and function while reducing immunosuppressive factors in the tumor microenvironment. VD also increases MHC expression, improving immune recognition, and interacts with signaling pathways such as PPARγ and PI3K/AKT/mTOR to modulate immune checkpoint molecules like PD-L1, offering potential targets for combination therapies.

## Peptide-based cancer vaccines

Peptide-based cancer vaccines have shown potential in HCC treatment by inducing targeted immunity TSAs. Glypican-3 (GPC3) is an HCC TAA that has been explored clinically as a therapeutic target. Sawada and coworkers conducted a phase I study of a GPC3-peptide vaccine in HCC patients and reported its association with specific immune responses without meaningful adverse reactions (194). In another phase I study, Greten et al. (195) investigated the immune effects of a multi-peptide vaccine (GV1001) containing several HCC-associated antigens [i.e., AFP, human telomerase reverse transcriptase (hTERT) and melanoma-associated geneA1 (MAGE-A1)] and found a reduction in Treg activity, but no detectable GV1001-specific immune response. Another phase I/II study by Loffler et al. (196) used a multi-peptide vaccine, consisting of 18 HLA-restricted peptides plus a TLR7/8/RIG agonist, and reported immune responses against at least 1 HLA-restricted antigen in 37% of patients. Despite these preliminary results, antigen heterogeneity in HCC represents a significant barrier and it has been suggested the addition of ICI may improve responses to peptide antigen-based cancer vaccines.

## Viral vector-based cancer vaccines

Based on viruses’ innate ability to infiltrate cells, the use of viral vector cancer vaccines represents a powerful method for delivering TSAs and triggering a cancer-specific immune response (197). Viral vector vaccines use modified viruses as delivery vehicles to transport tumor antigens into cells and stimulate an immune response. These vaccines can be engineered to express specific tumor antigens, effectively training the immune system to recognize and attack cancer cells. Adeno-associated virus (AAV) and adenoviral-based vectors are particularly attractive due to their large “gene packing” capacity, low pathogenicity, and high transduction efficiency. An AAV-based cancer vaccine has been reported to induce significant robust anticancer immunity in melanoma and colorectal cancer animal models, but no clinical studies have been conducted in HCC (198). Adenoviral-based personalized vaccines have been evaluated in melanoma patients demonstrating neoantigen specific and robust T cell responses (199). Instead of delivering TSAs, talimogene laherparepvec (T-VEC) vaccine consists of an oncolytic herpes virus-1 (HSV-1) engineered to deliver the granulocyte monocyte colony-stimulating factor (GM-CSF) gene to tumors, to elicit a strong immune response. Hecht and colleagues have recently reported preliminary results in 10 patients with liver cancers (4 HCC, 5 colorectal and 1 breast cancer and 6 non-HCC tumors) using intra-tumoral T-VEC injections. The investigators found that HCC had a lower baseline CD4^+^ CTLA4^+^ lymphocytes and Tregs, compared to non-HCC neoplasms. Additionally, HCC tumors were found with fewer infiltrating granzyme B^+^ CD8^+^ T cells after T-VEC treatment, compared to non-HCC neoplasms. Based on these results it was concluded T-VEC treatment was ineffective in HCC patients. An ongoing phase I study is exploring the efficacy of combining T-VEC with pembrolizumab in solid tumors, including HCC. Although viral vector-based vaccination has shown some potential in cancer patient, more clinical trials are needed to establish their role in the immunotherapy field.

## DNA and mRNA-based cancer vaccines

A phase I clinical trial assessed the safety and immunogenicity of a DNA vaccine encoding the HCC-associated peptide GPC3, demonstrating good tolerability and GPC3-specific immune responses in a subset of patients (194). Naked plasmid vaccine containing the AFP gene was examined in a mouse HCC model and it was reported that DNA vaccination was associated with protective immunity against Hepa1-6 cells, resulting in decreased growth of pre-established Hepa1-6 tumors in C57L/J mice (200). This study demonstrated the feasibility of delivering naked DNA plasmids encoding specific TSAs as a potential cancer vaccine strategy. However, additional work and novel designs are needed before this strategy could be successfully explored in the clinic. Messenger RNA (mRNA)-based vaccines have gained significant attention following their success during the COVID-19 pandemic. This technology offers the advantage of being easily customizable and works by delivering specific mRNA sequences encoding desire tumor antigens and instruct cells to produce these TAAs/TSAs in order to induce a robust anticancer immune response. Preclinical studies have shown AFP-mRNA vaccines can elicit CTL responses, providing protection against AFP-expressing HCC tumors, while others have reported AFP vaccination may result in M2 macrophage polarization (201, 202). Although application of mRNA vaccine technology is in its infancy, phase I studies are already being conducted in China exploring its promise in patients with HBV-related refractory HCC.

## Dendritic cell-based cancer vaccines

Dendritic cells (DCs) are specialized APCs that initiate and regulate immune responses by capturing tumor-specific antigens (TSAs) and presenting them to T cells. DC-based cancer vaccines involve isolating precursor monocytes, differentiating them into DCs *ex vivo*, loading them with TSAs or whole tumor lysates, and reinfusing them to elicit antigen-specific immune responses. Several clinical studies have evaluated DC vaccines in HCC for safety and efficacy. Tada et al. (203) tested a TAA-pulsed DC vaccine (AFP, GPC3, MAGE-1) in five advanced HCC patients, showing safety, TAA-specific T cell responses in all patients, and a clinical response in one patient. Another study administered similar DC vaccines to 12 HCC patients without residual tumors, reporting no recurrence in nine patients at 24 weeks, measurable T cell responses, and a median time to progression of 36.6 months versus 11.8 months in controls (HR = 0.41; *p* = 0.0031) (204). Lee et al. (205) conducted a randomized phase II trial in 156 post-treatment HCC patients, comparing six DC vaccinations to observation. No overall difference in recurrence-free survival (RFS) was observed; however, patients not previously treated with radiofrequency ablation (RFA) showed significant RFS improvement (HR = 0.49; *p* = 0.03), and baseline IL-15 levels correlated with prolonged RFS (HR = 0.16; *p* < 0.001). Palmer et al. (206) reported intravenous DC vaccines pulsed with HepG2 lysate in 35 HCC patients, achieving 28% tumor control over 3 months, with measurable T cell responses in several patients. A meta-analysis of 19 trials (1,276 patients) confirmed that DC vaccination enhanced CD4^+^/CD8^+^ T cell ratios, improved 1-year, 18-month, and 5-year PFS and OS (*p* < 0.05), with only mild adverse events (207). DC vaccines thus represent a promising immunotherapy for HCC, enhancing anti-tumor immunity and survival. Challenges remain, including selecting optimal TAAs/TSAs, standardizing DC production, defining responsive patient populations, and integrating DC vaccines into the broader immunotherapy landscape.

## Challenges in the development of cancer vaccines

Widespread clinical use of cancer vaccines faces many challenges, including selecting specific immunogenic TSAs, high costs, complex and unstandardized manufacturing, regulatory barriers, identifying ideal patient groups, and competition in immunotherapy. The biggest challenge is finding effective, specific antigenic targets, crucial for vaccine success. Cancer’s evolving mutations create neoantigens, expanding the antigenic landscape and enabling personalized vaccines tailored to individual tumors. Advances in digital medicine and AI are helping to quickly identify these targets, and integrating data mining, machine learning, and AI will be key to making cancer vaccines widely available.

## Adoptive cell therapy in HCC

Adoptive cell therapy (ACT) infuses immune cells, such as LAK, CIK, NK cells, TILs, or engineered T cells, expanded or modified *ex vivo* to target cancer cells, often following lymphodepleting chemotherapy. In HCC, autologous T cells expanded with CD3 and IL-2 reduced recurrence by 18% over 4.4 years (208). CIK-based ACT improved progression-free survival in a phase III trial (44 vs. 30 months) (209). TILs were feasible and safe in phase I studies (210), while NK cells kill tumor cells via cytokines, cytotoxic granules, and Fas-mediated apoptosis (211, 212). Ongoing trials are evaluating allogeneic NK-cell ACT in high-risk HCC patients (NCT02008929, NCT02854839). AFP is frequently expressed intracellularly and secreted by HCC cells, making it a rational target for TCR-based ACT. Four HLA-A2-restricted AFP epitopes have been identified in HCC patients (213), and objective remissions have been reported in a clinical trial using AFP-specific TCR T cells (NCT03132792). An early study evaluated HBV-specific TCR-expressing autologous T cells in advanced HBV-related HCC patients ineligible for liver transplantation. Treatment was well tolerated, with only 2 of 8 patients experiencing side effects. One patient achieved a partial response lasting 27.7 months, while others showed decreasing or stable HBV markers (214), demonstrating the feasibility of this ACT approach in HBV-related HCC. Advances in genetic engineering now allow modification of immune cells with synthetic receptors to enhance tumor antigen recognition. Most engineered ACT targets in HCC fall into three categories: tumor-associated or tumor-specific antigens (AFP, GPC-3), viral-derived antigens (HBV, HCV), and cancer-testis antigens (NY-ESO-1, MAGE). Transduced TCR (T-TCR) engineered T cells specifically recognize targeted tumor cells via MHC-restricted peptides. Intracellular TAAs/TSAs provide maximal cancer specificity and are accessible to TCR-based therapies, unlike other immunotherapeutic approaches. T-TCR cells can target intracellular antigens presented on HLA, but therapy is limited to patients with common HLA types (31, 215). T-TCR-based ACT has focused primarily on viral-associated peptides and AFP. Integration of HBV DNA into HCC cells allows production of HBV antigens recognizable by T cells, providing high-affinity TCRs for ACT development. Tan et al. (216) reported favorable safety and long-term benefit using short-lived mRNA HBV-T-TCR therapy in non-operable HBV-HCC. Similarly, initial data on autologous AFPc332 T-TCR T cells show promising results. Unlike conventional therapies, TCR-based ACT can offer durable protection, as engineered T cells may persist long-term and prevent cancer recurrence. Ongoing trials are investigating HBV, AFP, and MAGEA1 T-TCR cells (NCT03132792, NCT04368182, NCT03971747, NCT03441100). CAR-T cell therapy, another engineered ACT, has shown remarkable success in hematologic malignancies. CAR-T cells express synthetic constructs with an extracellular single-chain variable fragment, a transmembrane domain, a hinge region, intracellular signaling, and co-stimulatory domains, enabling potent, targeted antitumor activity (217, 218). Tumor-specific CAR-T cell therapy is an ACT not limited by MHC and can overcome immune escape (219, 220). CAR-T cells targeting HCC antigens include AFP, GPC3, CD133, HBV surface protein, EpCAM, and MUC1 (28, 29). GPC3, overexpressed in HCC but minimally in normal tissue, is the most common target (30, 221–224). Shi et al. (225) reported safety and antitumor activity of GPC3 CAR-T cells in GPC3^+^ HCC patients, and 11 phase I/II trials are ongoing, including one targeting GPC3/TGFβ (NCT03198546). CD133 CAR-T therapy in advanced HCC showed partial response in 1 of 21 patients and stable disease in 14, with median OS and PFS of 12 and 6.8 months, respectively (29). NKG2DL and EpCAM are also overexpressed in HCC, prompting their use as a CAR-T cell therapy targets (226, 227). NKG2DL and EpCAM are also being explored as CAR-T targets in ongoing trials (NCT05131763, NCT03013712).

Vitamin D modulates both T cell activation and immune suppression, potentially influencing the efficacy of adoptive cell therapy. While maintaining adequate vitamin D levels may benefit patients undergoing immunotherapy, the underlying mechanisms and optimal levels are still under investigation. Recently, Nath et al. (228) analyzed serum vitamin D levels in relapsed/refractory large B-cell lymphoma patients prior to anti-CD19 CAR-T administration and found that pre-CAR-T vitamin D insufficiency was independently associated with lower day-100 complete remission (CR) and overall survival (OS) rates. This study represents the first report linking vitamin D insufficiency to poorer clinical outcomes in CAR-T recipients. Further research is warranted to elucidate the mechanistic basis of this association and to explore the potential role of vitamin D supplementation in optimizing CAR-T therapy outcomes.

## Challenges for TCR and CART-based ACT

TCR-based therapies are limited to recognizing “matched” and relatively common, HLA alleles (such as HLA-A*02:01) to mount an appropriate anticancer response (15, 181). Other challenges include the potential for graft-versus-host disease (GVHD) and cost and complexity of generating large numbers of effector T cells consistently. CAR-T cell-based ACT also has its own challenges, including target antigen selection, manufacturing cost, degree of T cell transduction and *in vivo* expansion, need for lymphodepleting chemotherapy, and its association with a variety of potentially lethal side effects, including cytokine release syndrome, neurological dysfunction and prolonged bone marrow suppression, requiring hospital-bound monitoring during and after treatments. Thus, much work is needed to generalize the use of these novel and very promising ACT strategies in this cancer patient population.

## Immunotherapy and artificial intelligence

The intersection of cancer immunotherapy and artificial intelligence (AI) holds significant promise for advancing these rapidly evolving fields. AI technologies can accelerate the development of immunotherapy by predicting the immunogenicity of antigenic candidates, assessing their efficacy, and optimizing their composition to induce robust, cancer-specific immune responses. Current algorithms can analyze vast genomic, proteomic, and immunological datasets, enabling the identification of therapeutic targets with greater efficiency and supporting the development of personalized treatments, including antibody therapy, vaccines, and adoptive cell transfer (ACT) (229). Machine learning methods based on histopathology provide novel strategies for predicting immunotherapy response. Applications include immunohistochemical profiling, tumor-infiltrating lymphocytes (TILs), tumor-stroma ratio (TSR), and microsatellite instability (230). The advent of liquid biopsy further facilitates diagnosis and monitoring by detecting circulating tumor DNA, which AI algorithms can process to predict ICI efficacy (231). Biomarkers, such as circulating cytokines and tumor DNA sequences, may also help distinguish true progression from pseudo-progression (232). Advances in medical imaging now generate large-scale, high-resolution datasets beyond human interpretation. AI-based imaging analysis can reveal molecular and cellular features of tumors, allowing non-invasive evaluation of biomarkers to guide immunotherapy response (233–235). By integrating imaging, circulating biomarkers, and clinical parameters, AI enables monitoring of vaccine and ICI effectiveness and supports treatment adjustments. Beyond clinical use, AI can optimize research by analyzing experimental and real-world data to refine trial design and execution. Biopharmaceutical companies increasingly leverage AI to enhance vaccine and ACT development. Integrating multi-omics, radiomics, and clinical datasets into AI-based models offers a powerful approach for identifying patients most likely to benefit from specific interventions (236). Ultimately, AI-driven strategies are expected to improve the personalization, monitoring, and outcomes of cancer immunotherapies.

## Conclusion and future perspectives

HCC remains a major global health challenge with limited benefit from conventional therapies. Immunotherapy, particularly ICIs, has improved outcomes but is not curative. Combining ICIs with targeted agents shows promise in enhancing response rates and reducing relapse, yet patient outcomes remain highly variable. Resistance, driven by tumor heterogeneity, immunosuppressive cell recruitment, and onco-fetal niches, remains poorly understood (237–239). Identifying predictive biomarkers and functional assays is essential to stratify patients and personalize treatment. Combination therapies are under investigation, including ICIs with kinase inhibitors, anti-angiogenic agents, or local ablative methods, but the optimal timing, sequence, and pairing are unclear (240, 241). Careful optimization is needed to maximize benefit while limiting toxicity, particularly hepatotoxicity. Vitamin D is emerging as a potential modulator of the HCC immune microenvironment. While it demonstrates anti-tumor and immunoregulatory effects, its precise mechanisms, especially in synergy with ICIs, require clarification. Its influence on the gut microbiome, such as promoting *Bacteroides fragilis*, suggests possible systemic benefits, though clinical risks and advantages remain uncertain. AI offers new opportunities to refine therapy by integrating multi-omics, single-cell sequencing, patient-derived models, clinical data, and imaging (242, 243). Despite challenges in standardization and data integration, AI could guide personalized therapies and optimize clinical trial design, ultimately advancing HCC management.
